# Factors Influencing Anxiety Levels in Oncology Patients: A Study on the Impact of Earthquakes

**DOI:** 10.7759/cureus.57230

**Published:** 2024-03-29

**Authors:** Sule Karabulut Gul, Ahmet Fatih Oruc, Duygu Gedik, Muhammed Edib Mokresh, Omar Alomari, Mehmet Alper Kaya, Duygu Akincioglu, Huseyin Tepetam, Hakan Levent Gul

**Affiliations:** 1 Radiation Oncology, Kartal Dr. Lutfi Kirdar Training and Research Hospital, Istanbul, TUR; 2 Radiation Oncology, Istanbul Oncology Hospital, Istanbul, TUR; 3 Radiation Oncology, Hamidiye International School of Medicine, University of Health Sciences, Istanbul, TUR; 4 Neurology, Maltepe Medical Park Hospital Neurology Clinic, University of Istanbul Rumeli, Faculty of Sports Sciences, Istanbul, TUR

**Keywords:** psychosocial interventions, sociodemographic factors, cancer types, gender differences, psychological stress, earthquakes, oncology patients, anxiety

## Abstract

Objective: This study aims to explore the multifaceted factors influencing anxiety levels in oncology patients, with a specific focus on the impact of earthquakes in the context of Turkey. Our objective is to identify and understand sociodemographic, clinical, and lifestyle determinants associated with anxiety in cancer patients, examining how traumatic events, such as earthquakes, contribute to heightened anxiety levels.

Materials and methods: A cross-sectional study was conducted, involving 149 oncology patients undergoing treatment at two prominent oncology centers in Turkey. The study collected comprehensive sociodemographic information and assessed anxiety levels using the Beck Anxiety Scale. The dataset was analyzed using SPSS 20.0 (IBM Corp., Armonk, NY), employing a range of statistical methods including descriptive statistics, independent t-tests, Mann-Whitney U tests, and Chi-square tests.

Results: The findings underscore several factors significantly linked to anxiety levels in oncology patients. Notably, women, younger patients (age <65), and individuals with specific cancer types exhibited higher anxiety levels. Elevated anxiety was also associated with compromised physical functioning, experiences of earthquakes, irregular sleep patterns, dietary habits, fatigue, and the use of antidepressants.

Conclusion: This study provides insights into the intricate interplay of factors influencing anxiety levels in oncology patients. Understanding these determinants is paramount for tailoring effective psychosocial support and interventions. The results underscore the need for holistic approaches to enhance the overall quality of life for cancer patients. Gender, age, cancer type, physical well-being, lifestyle choices, and exposure to trauma all play pivotal roles in influencing anxiety levels. These findings hold practical implications for the development and implementation of targeted psychosocial interventions aimed at improving anxiety management and overall well-being for oncology patients.

## Introduction

Earthquakes, as significant natural disasters, not only pose threats to life and property but also impart profound psychological effects on individuals [1,2]. This impact is particularly pronounced in oncology patients who are concurrently navigating the complexities of a severe health condition [3]. The psychological well-being of individuals diagnosed with cancer is a critical aspect, given the concerns about disease recurrence and the ongoing process of psychological adaptation throughout their treatment journey [4].

Anxiety and depression experienced by cancer patients can substantially affect their quality of life, complicate treatment processes, and potentially influence life expectancy [5]. Understanding the specific context of earthquake anxiety in oncology patients becomes crucial, characterized by anxiety and fear responses occurring before, during, and after an earthquake [6].

Prior studies conducted in Turkey have underscored the considerable psychological impact of earthquakes on individuals [7,8]. Furthermore, anxiety and depression levels among cancer patients in Turkey were observed to surpass those in the general population [9]. Despite this, a notable research gap exists concerning earthquake anxiety levels among oncology patients in Turkey, a seismically active region with a historical record of significant earthquakes [1].

This study seeks to fill this gap by assessing anxiety levels in oncology patients following a major earthquake in Turkey. By investigating the psychological stress experienced by oncology patients in the earthquake's aftermath, we aim to enhance our comprehension of anxiety and earthquake anxiety in this specific population. The findings hold the potential to inform the development of more effective support and treatment strategies tailored to the unique needs of cancer patients confronting the dual challenges of cancer diagnosis and seismic events.

## Materials and methods

This cross-sectional study aimed to assess anxiety levels in oncology patients following the major earthquake on February 6, 2023, in Turkey. The study, after obtaining approval from the Kartal Dr. Lutfi Kirdar Research and Training Hospital Ethics Committee (approval number: 2023/514/254/42) and obtaining patient consent, examined sociodemographic information and anxiety levels in patients undergoing oncological treatment. Data collection occurred between March and September 2023.

Patients aged 18 and over who received oncological treatment from Kartal Dr. Lutfi Kirdar Research & Training Hospital and Istanbul Oncology Hospital in Istanbul, Turkey were included in the study. The two selected centers were chosen based on their geographical locations and the diverse patient populations they serve.

During the first stage of data collection (March-May), a sociodemographic information form was utilized, encompassing details such as age, gender, marital status, education level, occupation, income level, and illness duration. In the second stage (June-September), we assessed the patients' anxiety levels using the Beck Anxiety Scale (BAI). The scale's questions were administered in a face-to-face interview by a medical professional, and responses were recorded. The BAI comprises 21 items, each assessed on a scale from 0 to 3 for every item [10]. This scale gauges the anxiety symptoms experienced by the individual with a maximum total score of 63. The Turkish adaptation and the validation-reliability study for BAI were carried out by Ulusoy et al. [11].

Statistical analysis was conducted using the statistical software SPSS 20.0 (IBM Corp., Armonk, NY). Descriptive statistics were computed as frequencies and percentages. Mean and standard deviation values were calculated for continuous variables. To assess intergroup differences in BAI scores, we employed the independent t-test and Mann-Whitney U test for continuous data and used the Chi-square test to examine relationships among categorical variables. We established statistical significance at p < 0.05.

## Results

A total of 149 participants completed the survey, and their demographic information is presented in Table 1. The majority of respondents were male (58.4%, n = 149), and the mean age of the participants was 64.28 years (SD = 11.92, n = 149). Notably, 54.36% of the participants were aged 65 or older (n = 149). Marital status distribution was predominantly married (83.22%, n = 149), and educational levels varied, with the majority having completed primary school (57.05%, n = 149). Table 1 provides a comprehensive overview of the demographic composition of the study participants.

**Table 1:** Demographic characteristics of survey respondents. SD: Standard Deviation N: Sample Size

Variables		Data (N (%))
Age		Mean (SD):64.28 (11.92)
Age group	Age < 65	68 (45.64)
	Age ≥ 65	81 (54.36)
Gender	Male	87 (58.39)
	Female	62 (41.61)
Marital status	Single	5 (3.36)
	Married	124 (83.22)
	Widow	20 (13.42)
Educational level	Illiterate	8 (5.37)
	Primary school	85 (57.05)
	High school	30 (20.13)
	College	26 (17.45)
Cancer type	Head and Neck cancers	4 (2.68)
	Gastrointestinal cancers	14 (9.40)
	Lung cancer	24 (16.11)
	Breast cancer	33 (22.15)
	Other cancers	74 (49.66)
ECOG	ECOG (0, 1)	142 (95.30)
	ECOG (2, 3)	7 (4.70)
Stage	Early stage	14 (9.40)
	Advanced stage	96 (64.43)
	Metastatic	39 (26.17)
Presence of children	No	12 (8.05)
	Yes	137 (91.95)
Social security status	No	6 (4.03)
	Yes	143 (95.97)
Comorbidities	No	81 (54.36)
	Yes	68 (45.64)
Hometown	East	43 (28.86)
	South	9 (6.04)
	West	43 (28.86)
	North	54 (36.24)
Experienced earthquake	No	100 (67.11)
	Yes	49 (32.89)
Loss of relatives in earthquake	No	145 (97.32)
	Yes	4 (2.68)
Regular sleeping	No	52 (34.90)
	Yes	97 (65.10)
Regular diet	No	40 (26.85)
	Yes	109 (73.15)
Fatigue	No	69 (46.31)
	Yes	80 (53.69)
Occupation	Civil servant	28 (18.79)
	Tradesman	7 (4.70)
	Worker	30 (20.13)
	Housewife	42 (28.19)
	Self-employed	42 (28.19)
Residential area	Metropolitan city	141 (94.63)
	Village/Town	8 (5.37)
Living in a high-risk area	No	14 (9.40)
	Yes	135 (90.60)
Number of floors in the building	Detached house	10 (6.71)
	Less than 4 floors	58 (38.93)
	More than 4 floors	81 (54.36)
Taking antidepressants	No	121 (81.21)
	Yes	28 (18.79)

Moving on to anxiety levels, as detailed in Table 2, the majority of participants (81.9%, n = 149) reported experiencing mild anxiety, while 9.4% (n = 149) reported moderate anxiety, and 8.7% (n = 149) reported severe anxiety.

**Table 2:** Demographic comparison of anxiety score after the earthquake.

Variables		Anxiety score Mean (SD)	P-value
Gender	Male	5.24 (6.69)	< 0.001
	Female	12.71 (12.10)	
Age group	Age < 65	11.06 (11.98)	0.003
	Age ≥ 65	6.07 (7.30)	
Cancer type	Head and Neck cancers	3.00 (0.82)	< 0.001
	Gastrointestinal cancers	17.14 (14.05)	
	Lung cancer	8.63 (8.46)	
	Breast cancer	11.76 (12.31)	
	Other cancers	5.36 (6.91)	
ECOG	ECOG (0, 1)	7.85 (9.75)	0.005
	ECOG (2, 3)	18.57 (10.28)	
Experienced earthquake	No	6.93 (9.36)	0.018
	Yes	11.24 (10.71)	
Regular 7-9 hour sleep	No	15.25 (12.51)	< 0.001
	Yes	4.65 (5.60)	
Regular diet	No	12.38 (12.51)	0.013
	Yes	6.87 (8.51)	
Fatigue	No	4.12 (5.21)	< 0.001
	Yes	12.00 (11.61)	
Taking antidepressants	No	5.91 (8.23)	< 0.001
	Yes	18.89 (10.24)	

Analyzing anxiety scores by age group revealed a statistically significant difference (P = 0.003). Participants aged 65 and older exhibited lower anxiety scores (mean = 6.07, SD = 7.30, n = 76) compared to those under 65 (mean = 11.06, SD = 11.98, n = 73). This suggests that younger cancer patients tend to experience higher anxiety levels than their older counterparts.

Examining anxiety scores across different cancer types uncovered substantial variations (P < 0.001). Gastrointestinal cancers had the highest mean anxiety score (17.14, SD = 14.05, n = 35), while head and neck cancers had the lowest (3.00, SD = 0.82, n = 38). Notably, pairwise comparisons indicated significant differences in anxiety scores between specific cancer types.

Furthermore, a significant relationship was found between ECOG scores and anxiety levels (P = 0.005). Patients with higher ECOG scores (2 and 3) displayed significantly higher anxiety scores (mean = 18.57, SD = 10.28, n = 57) compared to patients with lower ECOG scores (0 and 1) with a mean anxiety score of 7.85 (SD = 9.75, n = 92).

The experience of an earthquake was associated with higher anxiety scores (P = 0.018). Participants who had experienced an earthquake had a mean anxiety score of 11.24 (SD = 10.71, n = 67), compared to 6.93 (SD = 9.36, n = 82) for those who had not.

Additional factors impacting anxiety levels included sleep patterns, diet, fatigue, and the use of antidepressants. Participants without regular seven to nine-hour sleep reported significantly higher anxiety scores (mean = 15.25, SD = 12.51, n = 91) compared to those with regular seven to nine-hour sleep (mean = 4.65, SD = 5.60, n = 58) (P < 0.001). Similarly, participants not following a regular diet had higher anxiety scores (mean = 12.38, SD = 12.51, n = 84) than those adhering to a regular diet (mean = 6.87, SD = 8.51, n = 65) (P = 0.013). Fatigue was strongly associated with elevated anxiety scores (P < 0.001), with fatigued participants reporting a mean anxiety score of 12.00 (SD = 11.61, n = 89), compared to 4.12 (SD = 5.21, n = 60) for those without fatigue. Finally, the use of antidepressants was linked to significantly higher anxiety scores (mean = 18.89, SD = 10.24, n = 37) in comparison to participants not using antidepressants (mean = 5.91, SD = 8.23, n = 112) (P < 0.001). These findings collectively emphasize the multifaceted nature of anxiety among cancer patients, influenced by demographic, medical, and lifestyle factors.

## Discussion

The present study sheds light on the intricate web of factors influencing anxiety levels in cancer patients, offering insights crucial for the development of targeted preventive and intervention strategies. Our findings align with prior research on gender differences [12], highlighting higher anxiety levels in female cancer patients and corresponding with suggested explanations involving hormonal disparities, social expectations, and coping strategy variations [13]. Similarly, the observed higher anxiety scores among younger patients resonate with previous studies [14], supporting the notion that older patients may exhibit more effective psychological adaptation and stress coping influenced by life experiences [15].

Our results also echo the significant impact of cancer type on anxiety levels, mirroring previous findings [16]. Patients with gastrointestinal or breast cancer experienced higher anxiety, likely due to the profound impact on their treatment process and quality of life, including more severe limitations in emotional and social functioning, as well as increased symptoms such as fatigue, dyspnea, insomnia, constipation (commonly associated with colorectal cancer), diarrhea (another common symptom in gastrointestinal cancer), and financial difficulties, as indicated by previous research findings [17]. Notably, participants with higher ECOG scores exhibited elevated anxiety levels, corroborating findings from Gotay et al. [18] and underscoring the connection between physical functionality and anxiety levels, particularly in patients with compromised physical functioning.

Moreover, participants with earthquake experience demonstrated higher anxiety scores, in line with Nakatani et al. [19]. Traumatic events, such as earthquakes, evidently influence the anxiety levels of cancer patients. Our findings further reinforce the impact of sleep, diet, and fatigue on anxiety levels, aligning with previous research [20], where regular sleep patterns and consistent diets were associated with lower anxiety, while high levels of fatigue correlated with elevated anxiety.

High levels of social support were linked to lower anxiety scores, underscoring the pivotal role of social support in stress coping [21]. Time since diagnosis emerged as a significant factor, with higher anxiety scores observed in participants diagnosed less than two years ago, consistent with Linden et al.'s [22] findings, suggesting that the duration of cancer diagnosis may influence anxiety levels.

In summary, these insights deepen our understanding of factors influencing anxiety levels, paving the way for tailored support and interventions to enhance the quality of life for cancer patients [23]. However, upon careful reevaluation of the original article, it is essential to acknowledge certain inherent limitations in this study. The sample size and representativeness of participants, although efforts were made to capture a diverse range, constrain the generalizability of findings. Future research with larger sample sizes is crucial for more reliable and generalizable results. Additionally, the limited scope of cancer types studied may restrict the applicability of results to patients with different cancer types. Future investigations should encompass a broader spectrum of cancer types and stages for a more comprehensive understanding. Furthermore, the absence of control groups limits the comparison of anxiety levels between cancer patients and healthy individuals. Future research incorporating such comparisons could offer valuable insights into the unique challenges faced by cancer patients and contribute to the development of effective anxiety management strategies. Despite these limitations, the insights gained from this study contribute to the broader understanding of anxiety in cancer patients and lay the groundwork for more extensive and inclusive investigations in the future.

## Conclusions

This study illuminates the intricate factors influencing anxiety levels in cancer patients, revealing key determinants such as gender, age, cancer type, ECOG score, fatigue, sleep, diet, and traumatic events. Women and younger patients experience higher anxiety, emphasizing the need for tailored support. Addressing lifestyle factors and psychosocial interventions can effectively manage anxiety and improve overall well-being. Future research should explore additional factors and involve diverse cancer types, paving the way for comprehensive strategies to enhance anxiety management and quality of life. This study establishes a robust foundation for future investigations in the field.
